# Chinese oral herbal paste for the treatment of stable chronic obstructive pulmonary disease: Protocol for a systematic review and meta-analysis

**DOI:** 10.1097/MD.0000000000016444

**Published:** 2019-07-12

**Authors:** Yan Zeng, Yu Li, Hua Wei, Chan Xiong, Li Liao, Ti-wei Miao, Bing Mao, Juan-juan Fu

**Affiliations:** a Department of Pneumology, Pidu District Hospital of Traditional Chinese Medicine, The Third Affiliated Hospital of Chengdu University of Traditional Chinese Medicine, Sichuan; b Respiratory Group, Department of Integrated Traditional Chinese and Western Medicine, West China Hospital, Sichuan University, Chengdu, China.

**Keywords:** Chinese oral herbal paste, chronic obstructive pulmonary disease, meta-analysis, protocol, systematic review

## Abstract

**Background:**

Chronic obstructive pulmonary disease (COPD) is a common chronic respiratory disease with high morbidity and mortality placing heavy social and economic burden. As a kind of complementary therapy for the treatment of stable COPD, Chinese oral herbal paste has been widely used and studied. The study aims to evaluate the clinical efficacy and safety of herbal paste in the treatment of stable COPD, and to provide evidence for its clinical application.

**Methods:**

We will electronically search databases, including Cochrane Central Register of Controlled Trials (CENTRAL), Web of Science, EMBASE, PubMed, Chinese National Knowledge Infrastructure (CNKI), WANFANG Database, Chinese Scientific and Technological Periodical Database (VIP), and Chinese Biomedical Database (CBM), from respective inception to June 2019 to collect randomized controlled trials (RCTs) of Chinese oral herbal paste for the treatment of stable COPD. The websites of Chinese clinical trial registry and international clinical trial registry, the reference lists of the retrieved articles, conference proceedings, and gray literature will also be collected. The quality of life, symptom scores, and exacerbation frequency will be measured as primary outcomes. Secondary outcomes include scores of traditional Chinese medicine (TCM) syndrome, clinical effective rates according to criteria in TCM, changes in lung function, 6-minute walking distance, and safety analysis. The Cochrane bias risk assessment and the GRADE method will be used to assess the quality of the original studies included. Merging analysis of data will be performed using Rev Man 5.3 software.

**Results:**

The systematic review will provide an evidence on the clinical efficacy and safety of Chinese oral herbal paste for the treatment of stable COPD, and will be submitted for publication in a peer-reviewed journal.

**Conclusion:**

The study will confirm whether Chinese oral herbal paste is an effective and safe intervention for the prevention and treatment of stable COPD.

## 1 Introduction

Chronic obstructive pulmonary disease (COPD) is a common respiratory disease that is characterized by persistent airway inflammation and airflow limitation that is mainly due to seriously exposure to toxic particles or gases.^[1]^ The global incidence and mortality of COPD is increasing.^[2,3]^ A national wide epidemiological survey has shown that the overall prevalence of COPD was 8.6%, namely, 100 million patients have COPD in China,^[4]^ which accounts for huge social and economic burden.^[5]^ In patients with COPD, the pulmonary functional decline and deterioration of quality of life is continuous and aggravated by acute exacerbations of the disease.^[6,7]^ In addition, various comorbidities, including cardiovascular disease, osteoporosis, anxiety, and depression, have severe impact on the course of disease which worsen the quality of life and increase mortality of the disease.^[8–10]^

Long-acting inhaled bronchodilators and/or corticosteroids are main pharmacotherapies for stable COPD.^[1]^ These pharmacologic therapies have been demonstrated to be effective in relieving symptoms and reducing future risk of exacerbations.^[11–13]^ However, persistent airway inflammation and frequent exacerbations remain exist in many patients which lead to deteriorated dyspnea and limitations in daily activities. In addition, the cost and adverse effects of pharmacologic therapies of western medicine still cannot be ignored.^[14–16]^ Low inhalation flow due to severely impaired lung function of the patients, presence of cognitive impairment, comorbidity, and, more importantly, patients’ compliance to medication all compromise the therapeutic effects of current inhaled treatments.^[17]^ Seeking other effective treatments for COPD to control symptoms and reduce exacerbation risk remains a challenging clinical problem.

**Figure 1:**
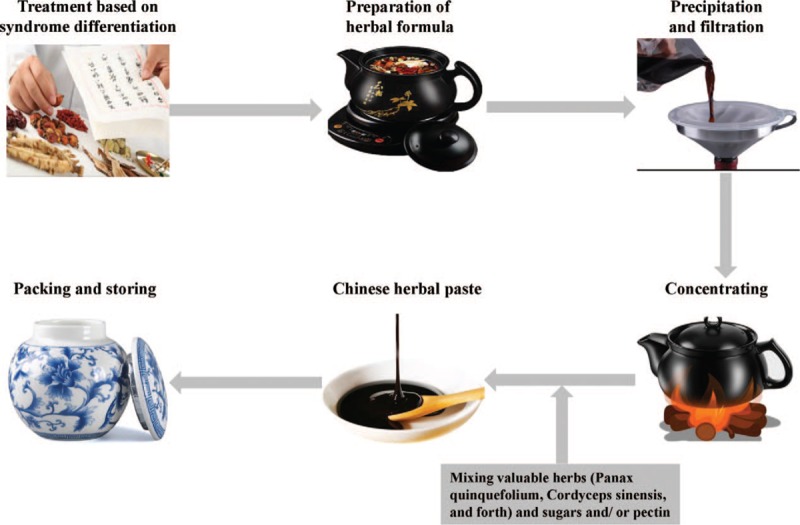
Process for making Chinese oral herbal paste.

Chinese herbal medicine (CHM), as one of alternative therapies for COPD, has become increasingly popular which has been used to treat symptoms of COPD, including chronic cough, dyspnoea, and sputum production, for thousands of years.^[18,19]^ COPD has been discussed and summarized as lung distention (fei zhang) according to traditional Chinese medicine (TCM) theory.^[20,21]^ CHM alone or integrated with routine western pharmacologic therapy has been widely used for the treatment of COPD in China,^[22]^ and been considered promising for improving symptoms, physiological impairment and comorbidities.^[23–25]^ The forms of CHM include decoction, oral liquid, powder, tablet, pill, paste, injection, and so forth.^[26]^ Chinese oral herbal paste is a kind of thick semiliquid or frozen dosage form of herbal formula which is made by concentrating herbal medicine decoction and adding auxiliary materials (Fig. 1). Oral herbal paste is believed to be effective in strengthening immunity, preventing the occurrence, development and recurrence of disease, and nourishing health, especially suitable for the treatment of chronic debilitating diseases.^[27,28]^ Due to low dosage, unique advantage in production methods, and the convenience for long-time usage of herbal paste, patients had better adherence to oral herbal paste.

An increasing number of randomized controlled trials (RCTs) have been conducted to explore the efficacy and safety of oral herbal paste on stable COPD and the results are promising.^[29,30]^ However, the quality of studies and the choice of clinical outcomes yielded different results, as such comprehensive and solid evidence is needed to support the clinical application of herbal paste. There is no published systematic review and meta-analysis to address this topic so far. Therefore, the current meta-analysis aims to evaluate efficacy and safety of oral herbal paste for stable COPD.

## 2 Methods

### 2.1 Registration

This systematic review and meta-analysis protocol have been registered on international prospective register of systematic review (PROSPERO) as CRD42019123715. The procedure of this protocol will be conducted in accordance with the Cochrane Handbook for Systematic Reviews of Interventions^[31]^ and the Preferred Reporting Items for Systematic Reviews and Meta-Analysis Protocol (PRISMA-P) statement guidelines.^[32]^

### 2.2 Inclusion and exclusion criteria

#### 2.2.1 Types of studies

Only RCTs that evaluated the effect and safety of Chinese oral herbal paste on patients with stable COPD will be eligible for this review, irrespective of blinding.

#### 2.2.2 Types of participants

Patients with a diagnosis of stable COPD will be included, regardless of sex, race, and settings, as diagnosed using any recognized diagnostic criteria, such as Global Initiative for Chronic Obstructive Lung Disease,^[1]^ or COPD guidelines in China.^[33]^ Stable COPD is defined that the symptoms such as cough, expectoration, and dyspnea are stable or mild, and the physical condition is maintained to the state before acute exacerbation. Studies with patients suffered from other respiratory system diseases in addition to COPD will be excluded.

#### 2.2.3 Types of interventions and comparators

The treatment group will be treated with Chinese oral herbal paste and conventional western medicine (WM). The control group will be treated with placebo oral paste and conventional WM, or conventional WM alone. Studies that included other TCM therapies such as acupuncture, decoction and granule of CHM, and so forth will be excluded. All the medications must be administered orally. There was no limitation on specific drug compatibility, doses and treatment duration.

#### 2.2.4 Types of outcome measures

The primary outcomes include quality of life assessed by the St George's Respiratory Questionnaire (SGRQ); symptom score represented COPD assessment test (CAT); and frequency of acute exacerbation of COPD within 12 months after study entry.

The secondary outcomes include scores of TCM syndrome based on symptoms of cough, sputum, dyspnea, wheeze, and self-care ability according to the Guiding Principle of Clinical Research on New Drugs of TCM^[34]^; clinical effective rates: clinical efficacy is graded into clinical cure, marked effectivity, effectivity, and no-effectivity according to the Guiding Principle of Clinical Research on New Drugs of TCM.^[34]^ Reduction rate of syndrome scores (n) is defined as the percentage change of the syndrome scores before and after treatment ((total syndrome scores before treatment − total syndrome scores after treatment)/total syndrome scores before treatment × 100%). Clinical cure is defined as n ≥90%, marked effectivity is defined as 70% ≤ n < 90%, effectivity is defined as 30% ≤n < 70%, and no-effectivity is defined as n < 30%. Clinical effective rates are calculated based on the grades of clinical cure, marked effectivity, and effectivity; lung function measured by changes of forced expiratory volume in 1 second; 6-minute walk distance; and safety analysis.

### 2.3 Data collection and analysis

#### 2.3.1 Search strategy

RCTs will be searched primarily in the Cochrane Central Register of Controlled Trials (CENTRAL), Web of Science, EMBASE, PubMed, Chinese National Knowledge Infrastructure (CNKI), WANFANG Database, Chinese Scientific and Technological Periodical Database (VIP), and Chinese Biomedical Database (CBM) from their respective inception to June 2019. Ongoing registered clinical trials will be searched on the websites of Chinese clinical trial registry (http://www.chictr.org.cn/) and international clinical trial registry (http://clinicaltrials.gov/). Additional trials will be searched by reviewing the reference lists of the retrieved articles, conference proceedings, and gray literature. The searching languages will include Chinese and English. The search terms or key words will be used alone or in varying combinations, as shown in Table 1.

**Table 1:**
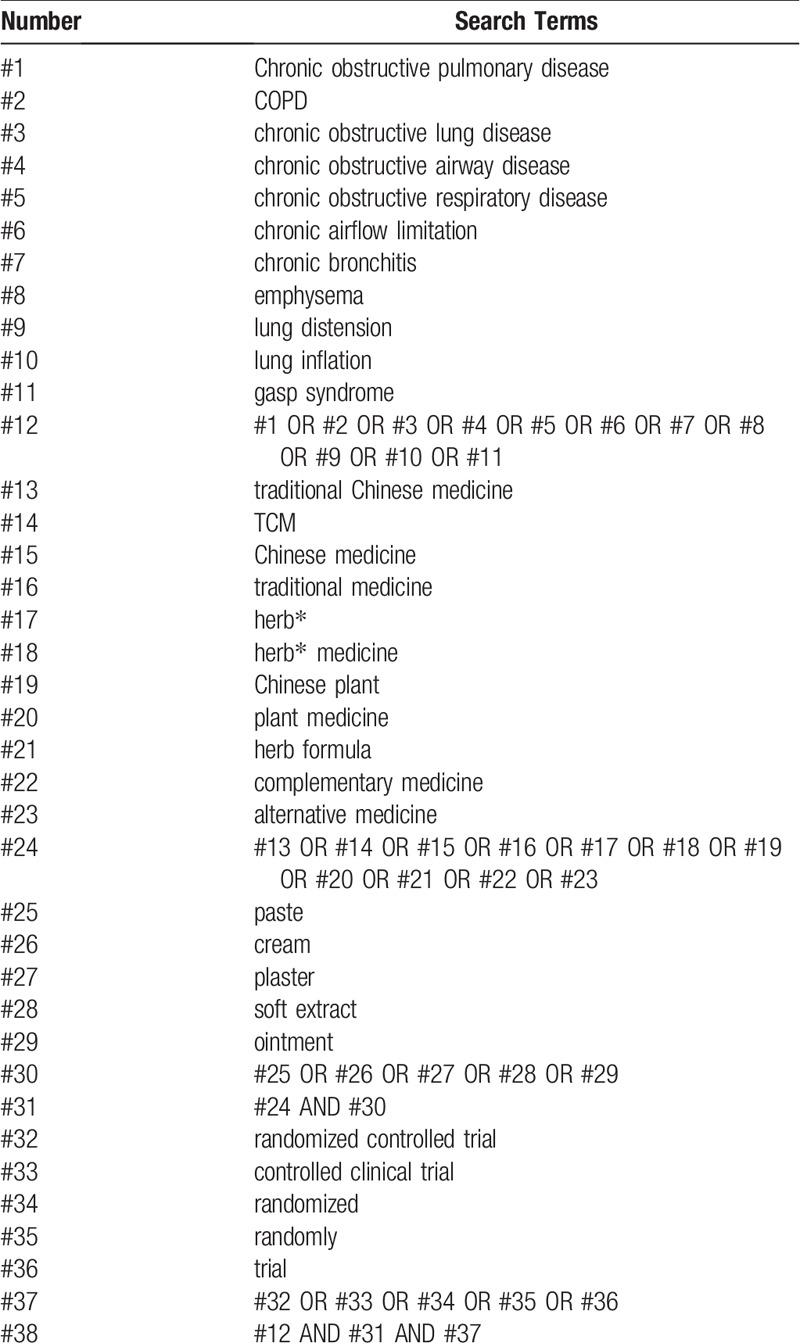
Search strategy for PubMed.

#### 2.3.2 Study selection and data extraction

The studies selected from all the databases will be integrated into Endnote X9 (Thomson Reuters). After removing duplicate trials, 2 reviewers (Yan Zeng and Yu Li) will independently review the titles and abstracts of each study to identify potential articles for further identification. Disagreements will be resolved through discussion with supervisors. All articles included will be reviewed by a third author (Juan-juan Fu).

**Figure 2:**
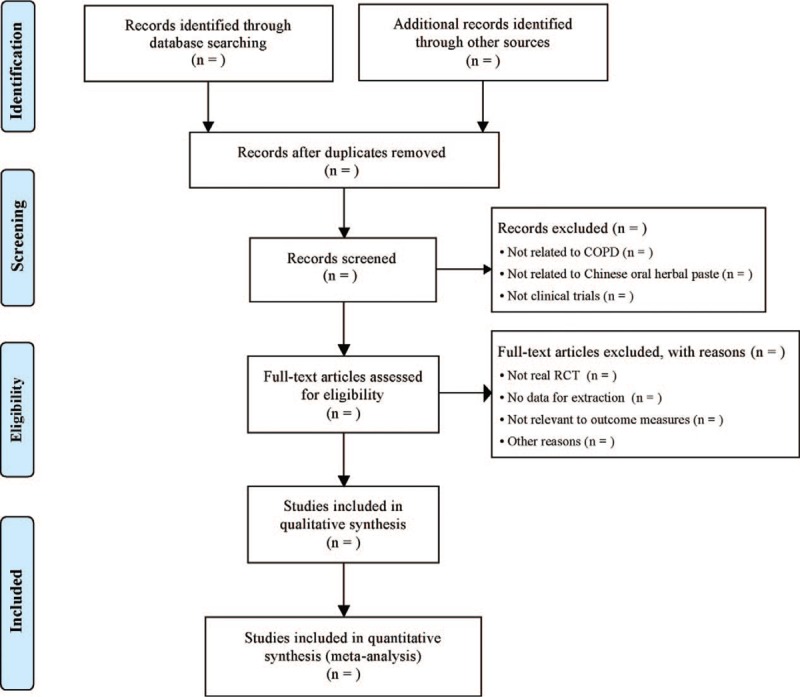
Study flow chart.

Two authors (Yan Zeng and Li Liao) will work individually to extract data into several standardized data collection forms, including study design (e.g., year of publication, study population, sample size, inclusion criteria, exclusion criteria, TCM syndrome, methodological information), patient characteristics (e.g., age, sex, lung function, course of disease), treatment and control interventions (e.g., administration methods, doses, duration of treatment, and follow-up), outcome measures, and adverse effects. All extracted data will be cross-checked for accuracy by the 2 reviewers. The lacking information will be retrieved by contacting the authors of the original studies, and trials will be excluded if information cannot be provided sufficiently. Discrepancy between reviewers will be resolved by group discussion. The whole process follows the PRISMA flow diagram, as shown in Figure 2.

#### 2.3.3 Quality assessment of included studies

The risk of bias will be assessed by 2 reviewers (Yan Zeng and Chan Xiong), which include selection bias (random sequence generation and allocation concealment), performance bias, attrition bias, detection bias, reporting bias, and other possible bias of all the included original studies according to the Cochrane systematic evaluation manual 5.1.0,^[31]^ and the disagreements will be resolved by consulting a third reviewer (Juan-Juan Fu) and group discussion.

Two researchers (Yan Zeng and Hua Wei) will use the Grades of Recommendation, Assessment, Development, and Evaluation (GRADE) method to evaluate the quality of evidences that include indirectness and imprecision of evidences, inconsistency of results, study limitations, and other potential bias between different studies of the same result.^[35]^ The GRADE system divides evidence into 4 grades: very low, low, moderate, and high. Each outcome will be assessed for the quality and importance from study design, risk of bias, inconsistency, indirectness, and imprecision and others.^[35]^

#### 2.3.4 Meta-analysis

Meta-analysis will be performed using Review Manager (Rev Man) 5.3 software provided by Cochrane collaboration. Mean difference (MD) will be used for statistical analysis of effect of continuous variables by Inverse-Variance method, and relative risk (RR) will be used for statistical analysis of effect of classified variables by Mantel-Haenszel method. Statistical significance of each effect size will be detected with 95% confidence interval (CI) and *P* value, and *P* < .05 will be considered statistical significance for all analyses. Interstudies heterogeneity among included trials will be tested by chi-square distribution test and *I*^2^ statistic. Fixed-effect model will be used for meta-analysis if no statistical heterogeneity among the results (*P* > .1 and *I*^2^ < 50%). When statistical heterogeneity exists among the results (*P*≤.1 and/or *I*^2^ ≥50%), the source of heterogeneity will be analyzed. Random-effect model will be used for further analysis after excluding the influence of significant clinical heterogeneity. If quantitative synthesis is not appropriate, only qualitative analysis will be performed.

#### 2.3.5 Subgroup analysis

If significant heterogeneity is detected between studies, we will explore whether the results are inconsistent in different subgroups. Simultaneously, the therapeutic principles of TCM syndrome, duration of treatment, severity of disease, measurements of results, age, sex, geographical area of the patients with COPD, and other different interventions will be taken into consideration to perform subgroup analysis.

#### 2.3.6 Sensitivity analysis

We will conduct sensitivity analyses of the primary results to explore the robustness of the review conclusions if feasible, after in consideration of impact of methodological quality, missing data, and sample size.

#### 2.3.7 Publication bias

When enough original studies are included (generally > 10 trials), publication bias analysis will be performed through funnel plot. Symmetrical funnel plot indicates low publication bias, otherwise high risk.

#### 2.3.8 Ethics and dissemination

The study does not require ethical approval because the original data are anonymous, which no privacy will be involved. And this study will eventually be published in a peer-reviewed journal in the form of a scientific paper.

## 3 Discussion

The primary therapeutic goals of COPD are to control symptoms and reduce future exacerbations.^[1]^ Acute exacerbations of COPD are significantly associated with decline of lung function, substantial morbidity, and mortality.^[36]^ Existing studies have shown that western pharmacologic therapies can reduce symptoms, the risk and severity of exacerbations, as well as improve the health status.^[37]^ However, many patients with COPD continue to experience cough, phlegm, distressing breathlessness, impaired exercise capacity, and frequent acute exacerbations despite receiving aggressive medical therapy such as triple inhaled medications according to current guideline.^[38]^ Chinese herbal decoction has been widely used in the treatment of diseases because of the highly individualized prescription, low cost, and less side effects. Evidence has shown the effectiveness of the decoction of TCM herbal formula on COPD.^[39,40]^ However, patients with chronic diseases have poor adherence to long-term herbal decoction due to the large volume of decoction, complex process of preparation, inconvenience in carrying and storing, and poor taste, which all affect the compliance and eventually the effectiveness of CHM.^[41]^

In TCM theory, lung dominates qi (the most essential substance that makes up the body and maintains life activities) and controls breathing. Long-term damage caused by exogenous pathogenic factors without appreciate treatments will lead to lung qi deficiency, by which the lung cannot govern qi dispersion, purification, and descending. If lung-qi fails to disperse and descend along with phlegm and blood stasis retention, resulting in symptoms of wheeze, cough, and dyspnea. Lung and spleen qi deficiency, lung and kidney qi deficiency, and qi and yin deficiency of lung and kidney are also common syndromes of stable COPD in TCM.^[42]^ The basic pathological features of lung distention (fei zhang) is exterior sthenia and interior asthenia,^[42]^ whereas the lung-qi deficiency is the most fundamental basis of COPD pathogenesis. Remarkably, patients with COPD are more likely to exacerbate due to lung-qi deficiency and exterior pathogenic cold in winter. The TORCH study, a large international multicenter COPD study, has demonstrated that COPD exacerbations and hospitalizations are more frequent in winter.^[43]^ Oral herbal paste is usually prepared and administrated during winter for 2 to 3 months. Compared with routine Chinese herbal decoction, herbal paste has the advantages of high drug concentration, bioavailability and stability, small volume, long storage time, satisfying taste, and convenience for carrying.^[44]^ Herbs that selected to make oral herbal paste are guided by the theories of “treatment based on syndrome differentiation” and “monarch, minister, assistant and guide” in TCM, aiming at balancing yin and yang, strengthening the self-healing capacity of the body to prevent and treat disease. Recent studies have reported that herbal paste is effective in relieving clinical symptoms, alleviating the decline of lung function, reducing the frequency of future acute exacerbation, and improving quality of life in stable COPD.^[45,46]^ Mechanisms studies have also been conducted in which the clinical effects of oral herbal paste may be associated with the enhancement of immunity and alleviation of inflammation and mucus secretion.^[47,48]^ However, the clinical efficacy and safety of oral herbal paste as another form of CHM with different administration method, dosage, and duration need to be evaluated by evidence-based medical research.

We will first assess the clinical efficacy and safety of Chinese oral herbal paste for the treatment of stable COPD by conducting a systematic review and meta-analysis. This meta-analysis will be carried out by several researchers strictly in accordance with the standard of PRISMA to guarantee the study quality. Compared with single clinical trial, this study will evaluate the efficacy and safety of Chinese herbal paste for COPD comprehensively, which will provide more credible evidence-based basis for its clinical application. The potential flaw of this study is that studies published in other languages instead of English and Chinese will be not included which may lead to publication bias. Meanwhile, different TCM syndromes and herbal formula among studies may also impact the interpretation of study results. We will conduct subgroup analysis based on TCM syndromes. The treatment principle of herbal paste is commonly based on asthenia syndrome and the herbal formula for COPD target lung-qi deficiency exclusively, and this will reduce the heterogeneity of study results. We will publish the report in a peer-reviewed journal in the form of a scientific paper through meta-analysis.

## Author contributions

**Data curation:** Yu Li, Li Liao.

**Formal analysis:** Yan Zeng, Bing Mao.

**Methodology:** Yan Zeng, Ti -wei Miao.

**Project administration:** Hua Wei.

**Resources:** Bing Mao, Juan-juan Fu.

**Software:** Yan Zeng, Ti-wei Miao.

**Visualization:** Chan Xiong.

**Writing – original draft:** Yan Zeng, Yu Li.

**Writing – review and editing:** Juan-juan Fu.
